# Direct Effect of Two Naphthalene-Sulfonyl-Indole Compounds on *Toxoplasma gondii* Tachyzoite

**DOI:** 10.1155/2013/716976

**Published:** 2013-10-21

**Authors:** Qasem Asgari, Hossein Keshavarz, Mostafa Rezaeian, Mohammad Hossein Motazedian, Saeedeh Shojaee, Mehdi Mohebali, Ramin Miri

**Affiliations:** ^1^Department of Parasitology and Mycology, Faculty of Health, Tehran University of Medical Sciences, Tehran 14177613191, Iran; ^2^Department of Parasitology and Mycology, Shiraz University of Medical Sciences, Shiraz, Iran; ^3^Medicinal and Natural Products Chemistry Research Center, Shiraz University of Medical Sciences, Shiraz, Iran; ^4^Department of Medicinal Chemistry, Faculty of Pharmacy, Shiraz University of Medical Sciences, Shiraz, Iran

## Abstract

Past studies have stated that the parasitostatic effect of IFN-*γ* is most likely due to the starvation of *Toxoplasma gondii* for tryptophan in the host cell. The aim of this study was to evaluate the direct effect of two new Naphthalene-Sulfonyl-Indole compounds as competitive molecules for tryptophan on viability and infectivity of *Toxoplasma* tachyzoites. Tachyzoites of RH strain were incubated in various concentrations (25–800 *μ*M) of 1-(naphthalene-2-sulfonyl)-2,3-dihydro-1H-indole and 1-[5-(2,3-dihydro-1H-indole-1-sulfonyl)naphthalene-1-sulfonyl]-2,3-dihydro-1H-indole for 1.5 hours. Then, they were stained by PI and analyzed by FACS. To evaluate the infectivity, 2 × 10^6^ tachyzoites exposed to the concentrations mentioned above were intraperitoneally inoculated into five mice from each group. Also, naïve parasites and parasites exposed to DMSO (control) were inoculated in both groups of mice. The LD_50_ of 1-(naphthalene-2-sulfonyl)-2,3-dihydro-1H-indole was 62 *μ*mol whilst the quantity of 1-[5-(2,3-dihydro-1H-indole-1-sulfonyl)naphthalene-1-sulfonyl]-2,3-dihydro-1H-indole was more than 800 *μ*mol. The infectivity of tachyzoites exposed to both of the compounds preserved and killed mice. No statistical correlation was seen between longevity of mice groups and different doses of the compounds. If we consider a well-organized transporter mechanism for indole compounds in the parasite, thus the designation of an antagonist that has indole groups can assist with the production of new drugs.

## 1. Introduction

Toxoplasmosis is an infectious disease caused by the intracellular protozoon parasite *Toxoplasma gondii*. This parasite can infect nucleated cells of birds and mammals, including humans. In humans, it causes severe medical complications in the fetus or in immunocompromised individuals [1].

Combination therapy with pyrimethamine and sulfadiazine is the standard treatment for toxoplasmosis [2]. Sulphadiazine is a dihydropteroate synthase (DHPS) inhibitor, while pyrimethamine inhibits dihydrofolate reductase (DHFR). Both enzymes are fundamental for the biosynthesis of pyrimidines in *T. gondii* [3].

Long-term treatment with these drugs can result in megaloblastic anemia or myelosuppression; consequently, folate supplementation should be used. Folate deficiency or an increased folate requirement might trigger the onset of myelotoxicity due to the use of these drugs [4]. Also, neutropenia and the potential teratogenic effects of pyrimethamine during the first trimester of pregnancy have been proven [5].

Current toxoplasmosis treatment for pregnant women is based on the administration of spiramycin in order to decrease the risk of fetal transmission. Grujić et al. have shown that even when greatly reducing residual infection, no spiramycin regimen can completely eradicate the parasite [6]. The common side effects of this drug include skin rash, itching, abnormal bruising, and uncommon gastrointestinal bleeding [7, 8].

Atovaquone, a structural analog of protozoan ubiquinone, has been shown to have significant activity against the bradyzoite stage of *Toxoplasma* in both *in vitro* and *in vivo* assays [9–12]. Conversely, Pearson et al. have shown that patients who administered atovaquone favorably responded to treatment, and their visual acuity stabilized or improved [13]. Unfortunately, there is strong evidence that the targeted parasites are able to spontaneously develop drug resistance by mutation of amino acid residues located in or near the atovaquone-binding site on cytochrome *b* [14].

Consequently, it is imperative to conduct a study on new and efficient drugs that have minimal side effects.

In the parasite, essential nutrients, which must be acquired from its host cells, can be considered as potential drug targets. One of the known essential amino acids is tryptophan used for intracellular proliferation of the parasite [15]. The parasitostatic effect of IFN-*γ* most likely results from the starvation of *T. gondii* for tryptophan [16]. Tryptophan is transferred across the cell membrane through facilitated diffusion with the aid of adenosine triphosphate (ATP) and specific proteins.

Tryptophan is present in most proteins and has an indole functional group as a distinguishing structural characteristic. Asai et al. have shown that the indole compounds as inhibitors of nucleoside triphosphate hydrolase can prevent tachyzoite replication *in vitro* [17].

We undertook the present study to assess the direct effect of two Naphthalene-Sulfonyl-Indole compounds on the viability and infectivity of* Toxoplasma* tachyzoites.

## 2. Materials and Methods

### 2.1. Compounds

**Figure 1:**
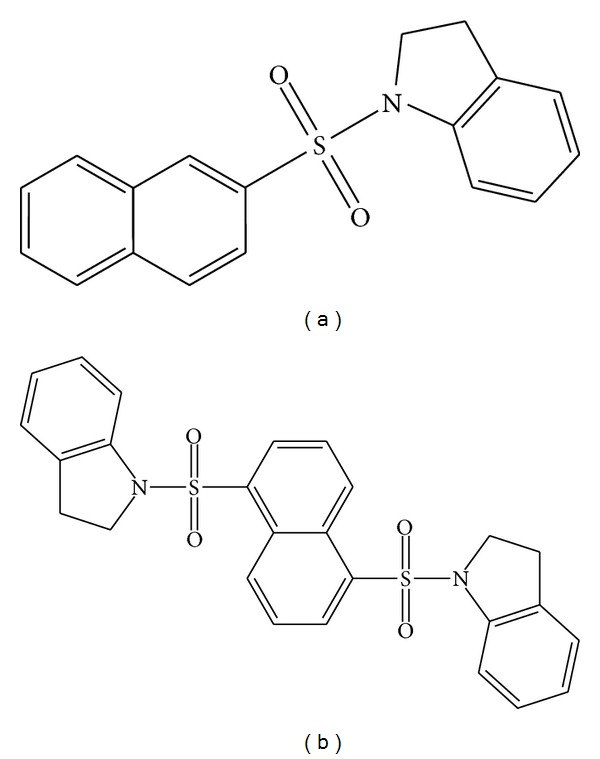
Formula of Naphthalene-Sulfonyl-Indole compounds: (a) [1-(naphthalene-2-sulfonyl)-2,3-dihydro-H-indole] and (b) 1-[5-(2,3-dihydro-1H-indole-1-sulfonyl)naphthalene-1-sulfonyl]-2,3-dihydro-1H-indole.

Compound A (Naphthalene-Sulfonyl-Indole). [1-(naphthalene-2-sulfonyl)-2,3-dihydro-H-indole], Compound Number; MolPort-000-556-394, Molecular Formula; C_18_H_15_NO_2_S, Molecular Weight: 309.4 (Figure 1).

Compound B (Naphthalene-Sulfonyl-diIndole). 1-[5-(2,3-dihydro-1H-indole-1-sulfonyl)naphthalene-1-sulfonyl]-2,3-dihydro-1H-indole, Compound Number; MolPort-001-637-694, Molecular Formula; C_26_H_22_N_2_O_4_S_2_, Molecular Weight: 490.6 (Figure 1).

### 2.2. Animals

 A total of 100 inbred BALB/c mice were provided from Pasteur Institute, Tehran, Iran, at 6–8 weeks aged and 22–25 gram weight. Animals were kept in the Laboratory Animal Center of Shiraz University of Medical Sciences, Shiraz, Iran. The procedures of all trials and sacrifices were identical for all animals. During the experiments, from April to May 2012, the animals were housed in cages and maintained under controlled environmental conditions (21 ± 2°C, 65–70% Room Humidity and a balanced diet with an access to food and water ad libitum). The experiments were undertaken based on the guidelines of the laboratory animals in the research and teaching book [18].

### 2.3. Parasites

The virulent RH strain of *T. gondii* was obtained from Tehran University of Medical Sciences, Tehran, Iran. Tachyzoites of the RH strain of *T. gondii *were maintained by serial intraperitoneal passaging in BALB/c inbred mice. After 72 hours, 10^6^ parasites inoculation in the mice, the tachyzoites were collected after repeated flushing of the peritoneal cavity by phosphate buffered Saline (PBS) at a pH of 7.2. Then, tachyzoites were harvested and centrifuged for 5 min at 200 g at room temperature to remove peritoneal cells and cellular debris. The supernatant was collected and centrifuged for 10 min at 1200 rpm (800 g) [19]. The pellet, enriched with parasite tachyzoites, was recovered with PBS and used in the experiments.

### 2.4. Extracellular Viability Assay

We dissolved the compounds in DMSO to obtain a final concentration of 10 mM. The final concentration of DMSO should not exceed 1%. Various concentrations (25, 50, 100, 200, 400, 800 *μ*M) of the compounds were then prepared by the following: 2.5–80 *μ*L of the final concentration was added to 920–997.5 *μ*L of solutions that contained 2 × 10^6^ tachyzoites per mL of PBS. Tachyzoites were incubated with either DMSO (as control) or the diluted compounds for 1.5 h at 4°C. Next, the tachyzoites were collected in Eppendorf tubes and incubated for 30 min at 4°C with 50 *μ*g/mL propidium iodide (PI, Sigma Company, USA). After incubation, the parasites were kept on ice until analysis. Positive controls for PI staining were acquired by incubating parasites in the presence of 0.2% saponin. The cell suspension was transferred into polystyrene flow cytometry tubes (BD Falcon Company, USA). We performed data acquisition and analysis, with a FACSCalibur flow cytometer (Becton-Dickinson, San Jose, USA) and Cell Quest Pro software. A total 1000–30000 event was acquired in the region that had been previously established as corresponding to the parasites.

### 2.5. Tachyzoite Infectivity in Animals

 A total of 2 × 10^6^ tachyzoites exposed to the concentrations of the compounds mentioned above were intraperitoneally inoculated into five mice from each group. For the control, naive parasites and parasites exposed to DMSO were intraperitoneally inoculated in both groups of mice. If the mice died, their liver touch smears were Giemsa stained and observed under light microscopy for parasite detection.

### 2.6. Data Analysis

Data were analyzed using SPSS software (version 11.5, Chicago, USA) by the Mann-Whitney nonparametric test. *P* < 0.05 was considered statistically significant.

## 3. Results

**Figure 2:**
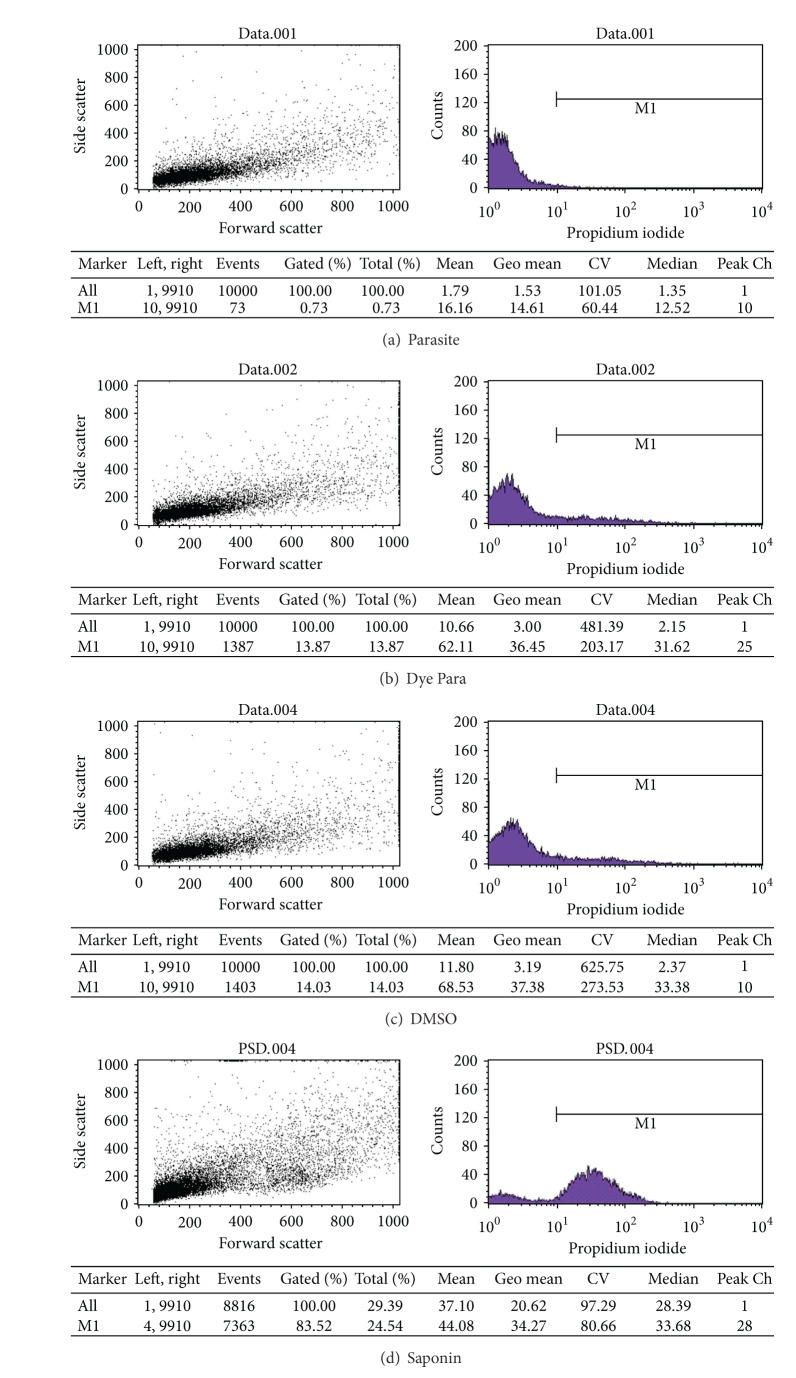

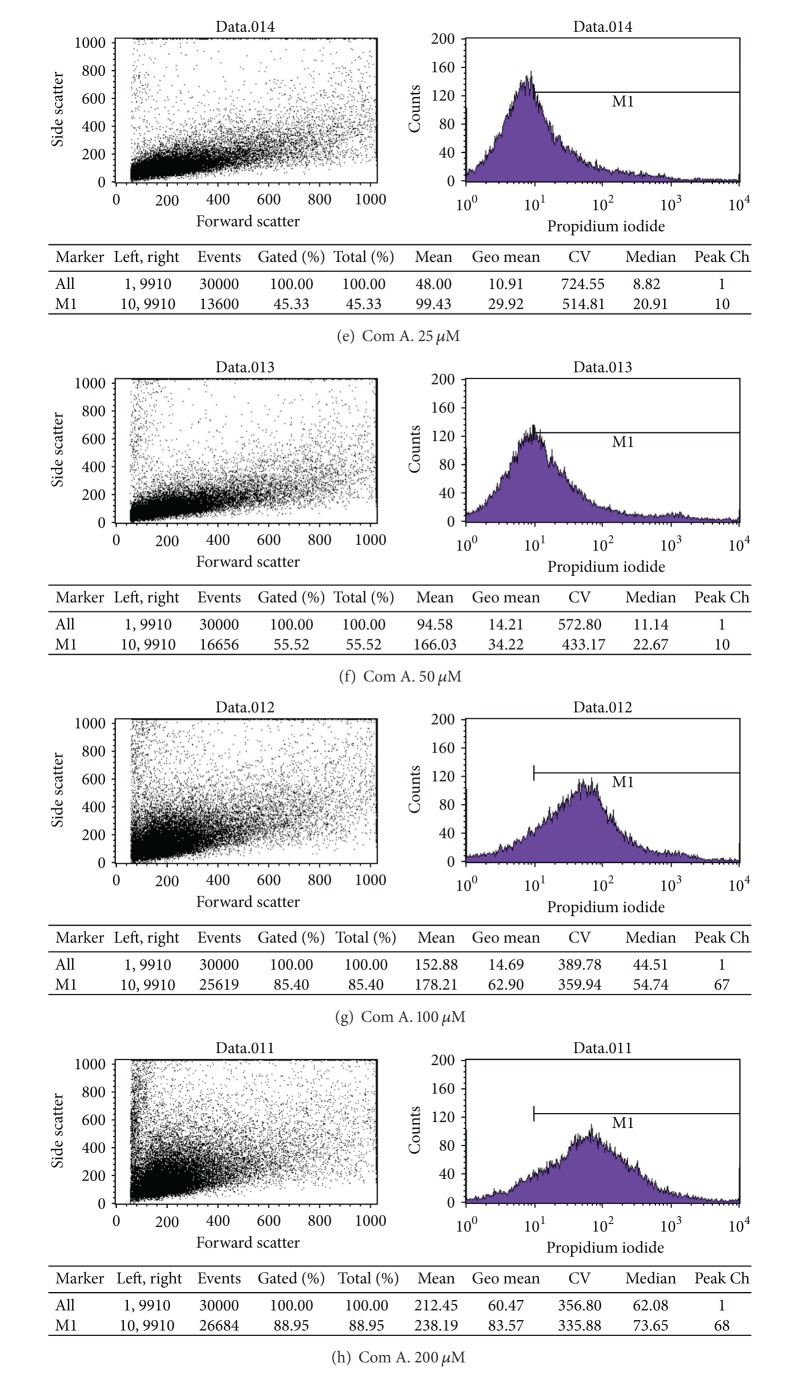

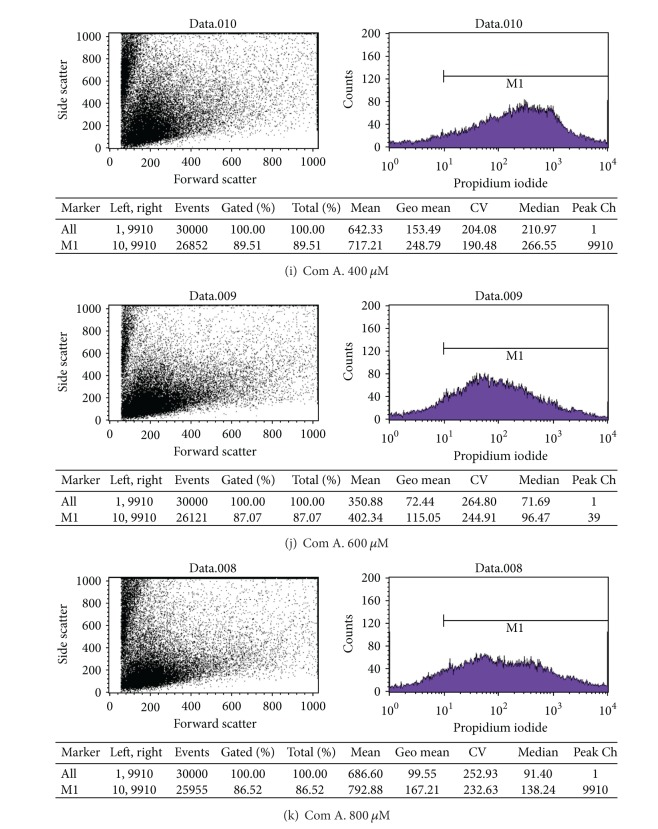
The result of flow cytometry tests on tachyzoite of *Toxoplasma* exposed to DMSO, saponin as positive control and different doses of compound A.

**Figure 3:**
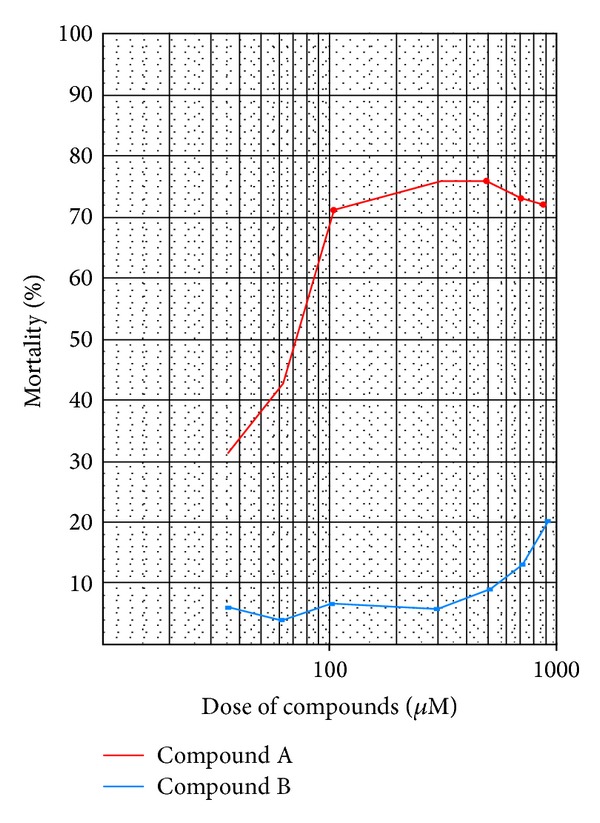
The mortality of tachyzoite of *Toxoplasma* exposed to compounds A and B.

 The result of flow cytometry tests on tachyzoite of *Toxoplasma* exposed to DMSO, saponin as positive control, and different doses of [1-(naphthalene-2-sulfonyl)-2,3-dihydro-H-indole] were shown in Figure 2. The LD_50_ of compound A [1-(naphthalene-2-sulfonyl)-2,3-dihydro-1H-indole] was 62 *μ*mol whilst the quantity of compound B [1-[5-(2,3-dihydro-1H-indole-1-sulfonyl)naphthalene-1-sulfonyl]-2,3-dihydro-1H-indole] was more than 800 *μ*mol. The mortality rate of tachyzoites exposed to the compounds was shown in Figure 3.

The infectivity of tachyzoites exposed to 25–800 *μ*mol doses of both of the compounds was preserved and these tachyzoites were able to kill the mice. The mean longevity (days) of mice infected by intact tachyzoites and tachyzoites exposed to the compounds was in the range of 5-6/6 (Tables 1 and 2). No statistical correlation was seen between the life duration of mice groups and different doses of the compounds.

## 4. Discussion

The treatment of toxoplasmosis is difficult due to the toxic effects of available drugs and the fact that reinfection may occur rapidly, so the introduction of new antitoxoplasma drugs and vaccines seems essential. Only a commercial vaccine (S-48), which is an attenuated *T. gondii *tachyzoite form, has been successfully employed for animal use. Vaccination with the live parasite cannot be safely carried out in humans [20]. The general consensus is that tissue cysts are resistant to commonly used drugs in the treatment of *Toxoplasma* infection, including pyrimethamine, sulfadiazine, and atovaquone, either alone or in a combination form. The cyst is believed to protect the parasite from the host immune system and act as a barrier for antiparasitic compounds [21].

Several studies indicated that the resistance to acute infection in mice is greatly related to endogenous IFN-*γ* [22, 23]. The activation of host cells against *Toxoplasma* induced by IFN-*γ* is dependent on the tryptophan concentration [24]. Däubener et al. have revealed that induction of indole amine 2,3-dioxygenase (IDO) contributes in antiparasitic mechanisms induced in human brain's microvascular endothelial cells by IFN-*γ* and TNF-*α*, which indicates that a protective activity is mediated by IDO [25]. Däubener et al. (2002) has shown that the depletion in intracellular tryptophan levels induced by IDO is an important mechanism by which IFN-*γ* controls the intracellular replication of *T. gondii* tachyzoites in various types of human cells [25]. IFN-*γ*-mediated induction of IDO appears to be critical for resistance of brain against *T. gondii* in human being [26].

The local tryptophan-depletion in microenvironments are assumed to be caused by macrophages, which have a unique high-affinity tryptophan importing system. The existence of highly specific and efficient transport machinery for tryptophan in macrophages has previously been confirmed. Macrophages can import and degrade tryptophan even at very low exogenous concentrations [27]. Tryptophan is an essential amino acid for the parasite's survival and proliferation in the host cell [15]. This amino acid has an indole functional group. Since *T. gondii* is an auxotroph for tryptophan and acquires the needed nutrients from its host cells. So, indole compounds can probably be introduced as drug antagonists.

Camalexin (3-thiazol-2′-yl-indole) was first isolated from the leaves of *Camelina sativa* in response to an infection by *Alternaria brassicae* [28]. Tsuji et al. [29] have reported the synthesis of camalexin by *Arabidopsis thaliana*, which is accumulated in high levels after infection with an avirulent strain of *Pseudomonas syringae*. Perhaps the indole ring of camalexin is derived from indole-3-glycerol phosphate group [28]. This is an intermediate molecule in biosynthesis of tryptophan [30, 31]. Another indole compound derived from tryptophan which is brassinin is provided from plants. The results of an investigation by Sellam et al. confirmed the antifungal effects of camalexin and brassinin at different developmental stages of both *Alternaria* species [32].

In this study, tachyzoites were exposed to the molecules while the parasites were intracellular and endured in the parasitophorous vacuoles (PV) of the host cells. The PV membrane is a permeable structure with a size prohibiting limit of ~1,300 Da [33]. Since *Toxoplasma* is auxotrophic for tryptophan and purine [15, 34], these pores may be used in receipt of the molecules using by host cytosolic ATP. An NTP hydrolase (NTPase) has been identified essential in the PV for tachyzoite replication within the host cells and may be partly responsible for this salvage process [35, 36]. It was shown that NTPases as new targets are still choices for chemotherapeutic measures against the disease. It seems that the enzyme is unique to the parasite and its activity appears to be imperative for the parasite's proliferation.

Modification in indole and phenol has shown that these compounds have modest IC_50_'s in the low micromolar ranges to inhibit *T. gondii* NTPases and prevent proliferation of tachyzoites [17]. Our study showed that the 1-(naphthalene-2-sulfonyl)-2,3-dihydro-H-indole was effective on the viability of tachyzoites. These experiments were undertaken on exposed tachyzoites, not intracellular ones. It seems that this compound affects tachyzoites based on mechanisms previously described [17].

It was demonstrated that indole naphthyridinones as inhibitors of bacterial enoyl-ACP reductases is a key enzyme in type II fatty acid biosynthesis (FAS-II) pathway and is a valid antimicrobial choice too [37]. The fatty acid synthesis of apicoplast in *T. gondii* is essential for organelle biogenesis and the parasite survival. Apicoplast prokaryotic fatty acid synthesis is a type II one and has recently received particular attention. The FAS II pathway is a metabolic process fundamentally different from the analogous FAS I pathway in humans that was recommended as a therapeutic measure [38, 39]. The effective levels of 1-(naphthalene-2-sulfonyl)-2,3-dihydro-H-indole on the viability of tachyzoites were less than other molecules. Our findings showed that the toxicity of compound A may not be related to the indole group. Alternatively, the membrane permeability of compound B may be low. In our study, the viability of tachyzoites exposed to different concentrations of 1-(naphthalene-2-sulfonyl)-2,3-dihydro-H-indole was different based on flow cytometry results and the survival of mice.

## 5. Conclusion

The presence of a well-organized transport system for indole compound within the parasite in conjunction with several effective mechanisms for the compound on *Toxoplasma* viability provide the chance for introduction of an antagonist material containing an indole group as a new drug.

## Figures and Tables

**Table 1:** The mean of life duration (days) of mice groups exposed to compound A: Naphthyl-Sulfonyl-Indole [1-(naphthalene-2-sulfonyl)-2,3-dihydro-H-indole].

Control	DMSO	Compound A 25 *μ*Mol	Compound A 50 *μ*Mol	Compound A 100 *μ*Mol	Compound A 200 *μ*Mol	Compound A 400 *μ*Mol	Compound A 600 *μ*Mol	Compound A 800 *μ*Mol
5.6	5.4	5.6	5.6	5.4	5	5.4	5.2	6

**Table 2:** The mean of life duration (days) of mice groups exposed to compound B: Naphthyl-Sulfonyl-diIndole or 1-[5-(2,3-dihydro-1H-indole-1-sulfonyl)naphthalene-1-sulfonyl]-2,3-dihydro-1H-indole.

Control	DMSO	Compound B 25 *μ*Mol	Compound B 50 *μ*Mol	Compound B 100 *μ*Mol	Compound B 200 *μ*Mol	Compound B 400 *μ*Mol	Compound B 600 *μ*Mol	Compound B 800 *μ*Mol
5.4	5.2	5.8	5.6	5	5	5.8	5.2	6.6
